# Retrospective Long-Term Comparison of Naturopathic Fasting Therapy and Weight Reduction Diet in Overweight Patients

**DOI:** 10.1155/2014/453407

**Published:** 2014-07-13

**Authors:** André-Michael Beer, Lena Elisabeth Ismar, Dominik Karl Wessely, Tanja Pötschke, Beate Weidner, Karl Rüdiger Wiebelitz

**Affiliations:** ^1^Department of Naturopathy, Blankenstein Hospital, Im Vogelsang 5-11, 45527 Hattingen, Germany; ^2^Department of Neurology, St. Johannes-Hospital, Springufer 7, 59755 Arnsberg, Germany; ^3^Clinic for Children and Adolescents, Prignitz Hospital, Dobberzinerstraße 112, 19348 Perleberg, Germany

## Abstract

In a follow-up study overweight and obese patients fasting according to Buchinger (modified) and a control group treated by a weight reduction diet in the context of an inpatient naturopathic complex treatment were compared using a questionnaire developed for a standardized phone interview 6.8 ± 1.1 years after inpatient treatment. During the inpatient treatment the fasting patients significantly more body weight, but at the time of the interview significantly more weight was gained again. 10.7% of the fasting patients and 31.9% of the control group lowered their weight at least 5% of their initial weight up to the interview. 42% of the fasting and 74% of the control group persistently changed their diet. The control group followed a significantly higher number of trained nutritional aspects. 21% of the fasting and 40% of the control group increased their leisure activity permanently. Continued improvement in quality of life was achieved by 16% of the fasting patients and 28% of the control group. The fasting therapy, carried out as part of the inpatient naturopathic complex treatment, turned out to be less suitable for the treatment of overweight and obesity compared to standard therapy. One likely determinant is the minor poststationary lifestyle modification.

## 1. Introduction

Multiple comorbidities are associated with overweight and obesity: cardiovascular diseases [1, 2], type II diabetes mellitus [3], and malignancies [4]. The moderately energy-reduced mixed diet (weight reduction diet) is considered as the standard treatment for obesity [5–7]. In view of the increasing prevalence of overweight and obesity fasting therapy, an established method of classical naturopathy [8, 9] appears to be a potential treatment option [10, 11]. Up to now only few studies examined the possible potential of fasting therapy. Therefore, scientific studies are urgently needed on this issue [12, 13]. While the weight loss during the period of fasting therapy is documented in several studies on different topics [14–16], studies that examine the long-term course of weight are rare. Whether fasting therapy leads to a long-term life style modification in obese patients, coupled with sustained weight reduction, a sustained changed diet and a lasting increase in physical activity and quality of life are not yet known. Likewise the long-term weight gain and lifestyle modification in overweight and obese patients after completion of fasting therapy versus standard weight reduction diet have not been scientifically evaluated. This question is therefore investigated in the present study.

A weight loss of 5% of the initial weight is generally considered successful and is also the defined treatment goal in the current guidelines for the prevention and treatment of obesity [6]. Therefore the primary outcome measure in this study is the proportion of patients who, after fasting therapy or weight reduction diet, achieved a sustained weight loss of at least 5% of their initial weight. The potential difference between the patient groups was determined.

Further examination criteria (secondary objectives) were group differences regarding the ongoing absolute weight reduction compared to baseline body weight, the weight loss of more than 2.25 kg as a further criterion of success, the weight development in poststationary continued weight reduction and/or prolonged weight maintenance, the persistent change in diet taking account of the rules of nutrition therapy, the observing of specific inpatient trained nutritional aspects and the relationship between the number of observed aspects of nutrition and weight loss, the ongoing increase in physical activity in the form of basic, leisure and sports activities, and the posthospital quality of life with regard to body weight.

## 2. Materials and Methods

The study was conducted as a single center comparative retrospective follow-up study. We compared both short- and long-term effects of the modified Buchinger fasting therapy [9] with the treatment with weight reduction diet in overweight and obese patients. The modified Buchinger fasting therapy, which includes vegetable stocks and vegetable juices, tea, and water, but no fruit juices or solid foods, was combined with regular defecation and exercise alternating with rest and was followed by a gradual return to solid food over 3 days. The mean duration of fasting therapy was 10.3 ± 1.8 days. 87% of fasting patients received subsequently a weight reduction diet and 13% a balanced basic diet [9, 17].

Weight reduction diet means a diet with reduced caloric feed charge. Patients receive a well-balanced low-fat and modified fat wholefood basic diet with a daily energetic deficit of 500–800 kcal. The food contains ~50% carbohydrates, ~30% fat, and ~20% proteins. Fat reduction is primarily achieved by restriction of animal fat leading to saturated fatty acids contributing less than 7% to the daily energetic supply. A high proportion of dietary fibers and complex carbohydrates ensure a prolonged feeling of satiety. The weight reduction diet patients continued their diet throughout the entire inpatient stay.

Both treatments were carried out in the context of inpatient naturopathic complex treatment. The mean duration of inpatient naturopathic complex treatment was 19.9 ± 1.9 days for all study participants.

The characteristics of the study participants as well as influences of inpatient naturopathic therapy were analyzed using the patient records and a standardized telephone interview. The questions explored the appraisal of fasting therapy, respectively, weight reduction diet, the development of body weight, diet, physical activity, and the influence on the quality of life as well as personal questions. Details and design of the questions, which were sent before the interview by mail to the patients, are displayed in “Supplementary Material” (see Supplementary Material available online at http://dx.doi.org/10.1155/2014/453407). These questions were then asked in the phone interview. The question set for the weight reduction diet patients was the same, with the replacement of “Heilfasten” by “Reduktionsdiät.”

The Ethics Committee of the Ruhr University Bochum approved the study (registration number 2976, May 16, 2007), which was undertaken according to the ethical principles of the Declaration of Helsinki.

All patients were sent written information about the study, the planned phone interview, and the voluntariness of participation. They gave oral consent at the beginning of the phone interview.

Patients aged 20 to 70 years who had undergone from 1999 to 2002 as part of the inpatient naturopathic complex treatment at the Blankenstein Clinic, Hattingen, a fasting therapy or a treatment with a weight reduction diet and were overweight (BMI ≥ 25 kg/m²) or obese (BMI ≥ 30 kg/m²) at this time were included.

Excluded from the study were all patients who had at the time of inpatient therapy one of the following diagnoses: hepatic or renal failure, decompensated hyper- or hypothyroidism, diabetes mellitus type 1, malignant tumors, Crohn's disease or ulcerative colitis, dementia, eating disorders such as anorexia nervosa, bulimia, or binge eating disorder, drug addiction, or serious psychiatric disorders. Also excluded were pregnant or breast feeding women and patients taking medications for overweight and obesity and patients after bariatric therapy.

The record search identified 728 fasting patients and 252 patients who had received a weight reduction diet. 367 of the fasting patients (50.4%) and 75 patients with weight reduction diet (29.8%) met the inclusion and exclusion criteria. Of the 367 fasting patients a random sample of 200 patients generated using the statistical program SPSS and all 75 patients with weight reduction diet were selected for the telephone interview.

At the time of the telephone interview the exclusion criteria were retrieved and reapplied. The reasons for the subsequent exclusion of 78 fasting and 28 weight reduction diet patients are shown in Table 1.

**Figure 1:**
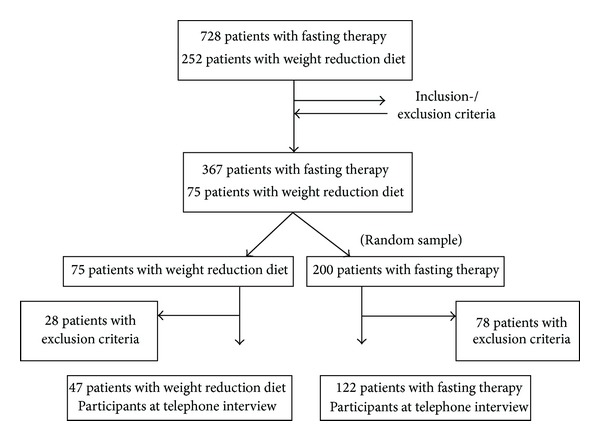
Recruitment of study participants.

122 of the 200 fasting patients (61%) and 47 of the 75 weight reduction diet patients (62.7%) ultimately participated in the phone interview. The phone interview was performed on average 6.8 ± 1.1 (standard deviation) years after the inpatient therapy (see Figure 1). The mean age of the fasting patients was 55.7 ± 7.4 years on admission and 54.5 ± 10.9 years for the weight reduction diet patients.

Demographic and biometric characteristics of the patients in the study are shown in Table 2.

Statistical analysis was performed using the computer program “SPSS 17.0 for Windows.” Arithmetic mean, standard deviations, medians, and absolute frequencies and percentages were calculated. The presence of normal distribution of metric data was checked using the Kolmogorov-Smirnov test. Statistical tests for parametric data were performed using Student's *t*-test and the Mann-Whitney *U*-test. For nominal and ordinal data Pearson's chi-square test and Fisher's exact test were applied. If the significance level (*P*) is less than the significance level *α* = 0.05, the result is called statistically significant. If it is less than the significance level *α* = 0.01, the result is called statistically highly significant [18]. Statistical tests were performed two-tailed in all cases.

Additional descriptive ANCOVAs were performed for BMI and weight with the posttreatment, respectively, actual variable as dependent variable, the group as fixed factors, and pretreatment score and other meaningful and influencing variables as covariates.

As part of the literature review searches in MEDLINE using PubMed and in the databases AMED, EMBASE, CCMed, and CAMbase with the help of the DIMDI were performed because most scientific literature regarding naturopathy is included in these databases. Additionally the Internet search engines Google, Altavista, and Metager were used.

## 3. Results

The fasting patients reduced their body weight from 86.0 ± 14.1 kg on admission to 80.1 ± 13.2 kg at the end of the fasting therapy. This is a statistically highly significant weight loss during the inpatient fasting therapy (*P* < 0.001).

At the telephone interview the average weight of the patients was 86.5 ± 15.6 kg and was thus 0.5 kg above the mean initial weight on inpatient admission. While the weight regain between the completion of fasting therapy and the telephone interview of the fasting patients was statistically highly significant (*P* < 0.001), there was no difference between the initial weight and the weight at the phone interview (*P* = 0.349).

**Figure 2:**
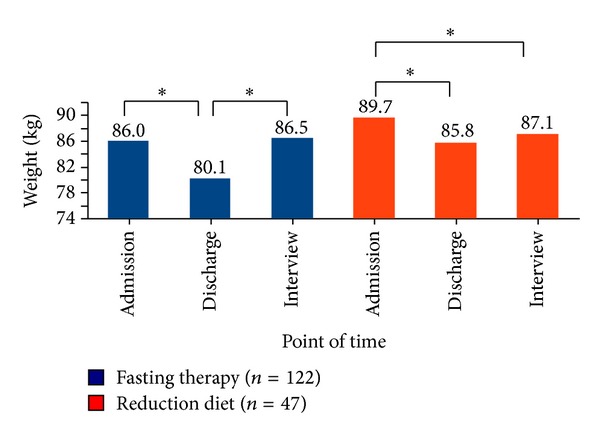
Mean of body weight (kg) at the beginning of inpatient treatment, end of treatment, and at telephone interview. *Significant difference within the treatment groups.

The weight reduction diet patients reduced their body weight during the inpatient treatment from 89.7 ± 14.3 kg to 85.8 ± 13.5 kg at the end of the therapy. Again the weight reduction between admission and the end of treatment (*P* < 0.001) is statistically highly significant. The average rebound of the weight up to the telephone interview was 87.1 ± 16.8 kg, making it an average of 2.6 kg below the initial weight and 1.4 kg above the weight at the end of the therapy. The mean body weight at the time of the interview differed statistically significantly from baseline body weight (*P* = 0.029), but not significantly from the weight at the end of the inpatient treatment (*P* = 0.232) (Figure 2).

**Figure 3:**
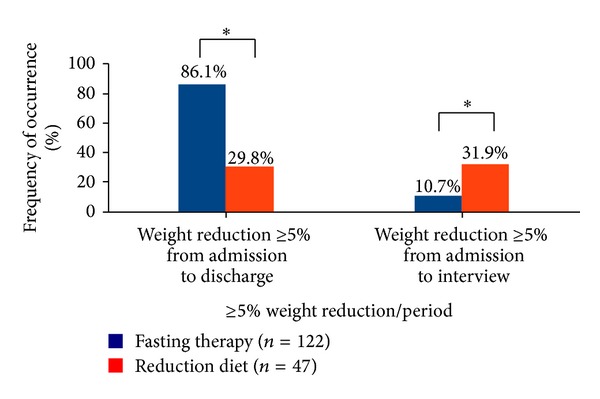
Frequency of weight reduction ≥5% of initial weight at the end of inpatient treatment, respectively, at the telephone interview. *Significant differences between the groups (*P* < 0.001).

During inpatient therapy the fasting patients reduced on average significantly more body weight than the weight reduction diet patients (−5.9 ± 1.9 kg versus −3.9 ± 1.7 kg, *P* < 0.001) but increased significant more weight again up to the interview (+6.4 ± 6.3 kg versus +1.4 ± 7.7 kg, *P* < 0.001). Overall 10.7% of the fasting patients and 31.9% of the weight reduction diet patients lost at least 5% of their initial weight (*P* < 0.001), the difference being a small to medium statistical effect (*w* = 0.26). On the whole a significantly greater proportion of the weight reduction diet patients (47%) than of the fasting patients (12%) continued the weight reduction after inpatient treatment until the time of the interview (see Figure 3).

Considering besides the 5% threshold an absolute weight reduction of more than 2.25 kg from baseline body weight as a successful weight loss, 26.2% of the fasting and 55.3% of the weight reduction diet patients fulfilled this criterion, which is a statistically highly significant (*P* < 0.001) difference between the groups with a small to medium statistical effect (*w* = 0.28).

**Figure 4:**
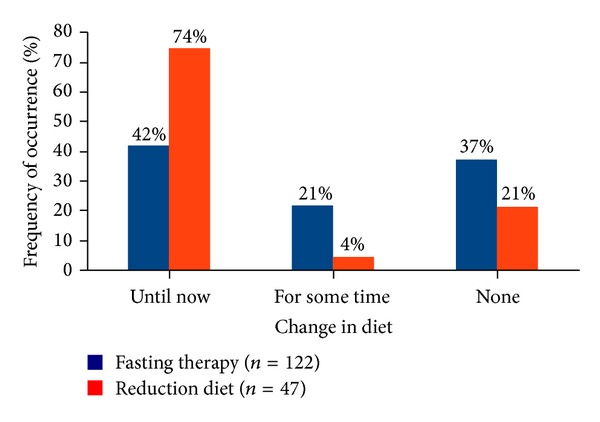
Frequency of dietary changes after inpatient therapy. Significant difference between the groups (*P* < 0.001).

Looking at the postdischarge dietary changes to a healthy diet according to the requirements of nutrition therapy after discharge from hospital, a clear and statistically highly significant difference between the groups was found (*P* < 0.001). About three quarters of the weight reduction diet patients (74%) and less than half of the fasting patients (42%) changed their nutrition after the inpatient therapy “up to this day,” that is, up to the time of the interview (see Figure 4).

**Figure 5:**
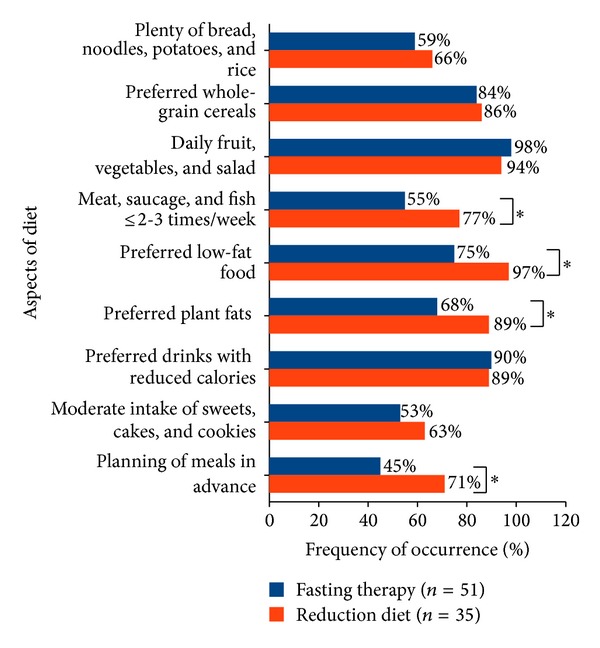
Frequency of taught dietary aspects, which study participants observed who changed diet after inpatient therapy. *Significant differences between the groups.

Of these participants the weight reduction diet patients followed a significantly higher number of inpatient trained nutritional aspects (*P* = 0.001). Thus 77% of the weight reduction diet patients versus 55% of the fasting patients stated to consume meat, sausages, and fish at most two to three times per week (*P* = 0.042). 97% of weight reduction diet patients versus 75% of fasting patients preferred low-fat foods (*P* = 0.006). Furthermore, 89% of the weight reduction diet patients and 68% of the fasting patients indicated preferring vegetable fats (*P* = 0.023). Finally, 71% of the weight reduction diet patients and 45% of the fasting patients planned their meals at least a week in advance (*P* = 0.026) (Figure 5).

**Figure 6:**
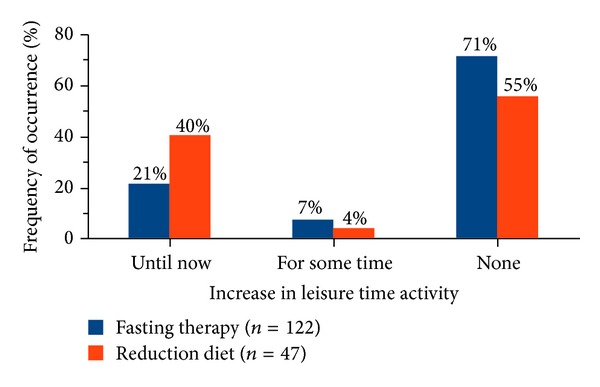
Frequency of increased leisure time activity after inpatient therapy. Significant difference between the groups (*P* = 0.041).

While the base and the sports activity was increased in both groups poststationary at similar rates (*P* = 0.707 and *P* = 0.336), the increase of leisure time activity was significantly different between the groups (*P* = 0.041). 21% of the fasting and 40% of the weight reduction diet patients increased their leisure activity persistently (Figure 6).

**Figure 7:**
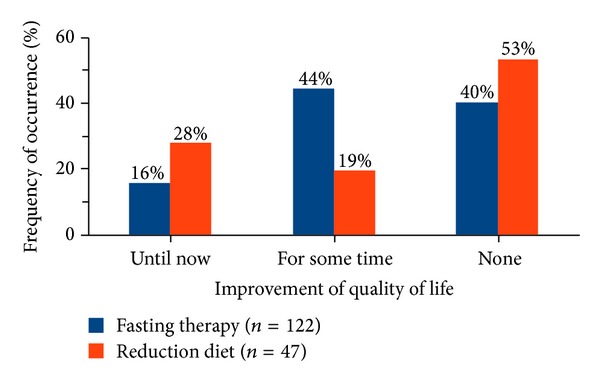
Frequency of improvement of quality of life regarding body weight after inpatient therapy. Significant difference between the groups (*P* = 0.008).

The sustained improvement of quality of life in relation to body weight also showed a significant difference (*P* = 0.008) between the groups. 16% of the fasting and 28% of the weight reduction diet patients experienced a sustained improvement in quality of life in this regard (Figure 7).

Additional descriptive ANCOVA for BMI, respectively, weight with the actual measurement as dependent variable and the treatment group as fixed factor shows significant results for all covariates pretreatment score (*P* < 0.001), negative concomitant conditions (sum of “yes”-answers to “no support by family/social contacts,” “stressful everyday life,” “immobility due to disease,” “medication impairing weight loss,” “eating because of chronic pain,” “dramatic biographical events like death of a family member,” “stopping smoking,” “healthy food not tasty,” “difficulties to reduce eating candies and cakes,” and “other factors”; *P* = 0.002, resp., *P* = 0.005), physical activity (sum of increased everyday life activity, estimated with half an hour per day, leisure time activity, time used for sports, time used for gardening, time used for walking and biking, time for ascending stairs, 2 minutes per floor; *P* = 0.003, resp., *P* = 0.014), and number of taught nutritional aspects applied in everyday life (*P* < 0.001). Only the test between the treatment groups is not significantly different in this ANCOVA (*P* = 0.440, resp., *P* = 0.692). If the covariate “number of taught nutritional aspects applied in everyday life” is omitted, the test between the treatment groups in the ANCOVA turns to significance (*P* = 0.003, resp., *P* = 0.010) showing that the difference between the treatment groups concerning this factor is mainly responsible for the different success rates in the treatment groups.

Descriptive ANCOVAs for BMI and weight with the measurement after treatment at discharge from hospital as dependent variable and the treatment group as fixed factor show significant (for weight and negative concomitant conditions only tendential) results for the covariates pretreatment score (*P* < 0.001) and negative concomitant conditions (*P* = 0.031, resp., *P* = 0.060) and the test between the treatment groups (*P* < 0.001).

ANCOVAs with additional covariates as age, age at manifestation of adipositas, duration of adipositas in years, adipositas of family members, and number of educational teaching sessions show no additional significant factors (the significance of the covariates described above is partly lost when adding too many other covariates in the model).

## 4. Discussion

A sustained weight loss of 5% of the initial weight is the primary goal of therapy according to the guideline for the treatment and prevention of obesity [6], because this is considered to be an achievable goal and also implicates health benefits. In this study, 10.7% of the fasting and 31.9% of the weight reduction diet patients reached this goal. While participants after weight reduction diet had good long-term results compared to current literature, the fasting patients reached on average their initial weight again after an initial weight loss. The small to medium statistical effect with respect to the difference between fasting and weight reduction diet patients appears to be clinically relevant against the background of low long-term success rates in the treatment of obesity. Furthermore, the fasting patients achieved unlike the weight reduction diet patients no lasting weight reduction of at least 2.25 kg of the starting weight, which would be beneficial for health. The reduction of cardiovascular risk factors and an increase in health and well-being have been demonstrated in various studies [19] for a permanent weight loss of 2.25 kg. One likely determinant of the less successful long-term weight loss of fasting patients constitutes the lesser lifestyle modification in this group.

Regarding the development of the weight after discharge from hospital only 12% of fasting patients compared with nearly 47% of weight reduction diet patients were able to continue the reduction of body weight after the end of inpatient treatment. In addition 14% of fasting patients and 49% of weight reduction diet patients gained persistent weight stabilization. One possible explanation for this comparatively high proportion of fasting patients who could not stabilize their weight after discharge from hospital is that fasting lowers plasma glucose levels which on the other hand may result in subsequent weight regain [20].

In both groups most study participants who could not stabilize their body weight increased their weight again within the first 4–12 months after the inpatient treatment. In the context of current literature on the treatment of obesity a weight regain after about 5 months turns out to be a typical phenomenon [21].

Finally 42% of fasting patients and 74% of weight reduction diet patients changed their diet after discharge from hospital up to the time of the telephone interview according to the rules of nutrition learned during the inpatient therapy (Figure 4). A prevailing proportion of patients with successful weight reduction could only be detected in the group of study participants who had changed their nutrition for the whole observation period.

But this study showed not only differences between fasting and weight reduction diet with respect to the proportions of patients who switched their diet, but also detailed changes in nutrition (Figure 5). While study participants following up to five aspects of nutrition did not achieve weight loss (median), study participants following eight aspects of nutrition reduced their weight (median) for 4.7 kg and study participants following nine aspects lost minus 13.0 kg body weight (median). With respect to individual aspects of nutrition the weight reduction diet patients ate significantly less meat, sausage, and fish compared to the fasting patients and preferred significantly more low-fat foods and vegetable fats. Thus, weight reduction diet patients reduced the amount of dietary fat significantly more than the fasting patients. This may partly be due to a lower consumption of meat and sausage. The National Nutrition Survey in Germany in 2008 showed that the majority of recorded dietary fat is caused by the consumption of meat and sausage [22]. Moreover, the weight reduction diet patients significantly more often planned their meals in advance for the following week. The planning of meals can be understood as a kind of cognitive control of eating habits [5] and proved in literature as an effective strategy for long-term successful weight loss [23].

One possible explanation for the fact that the fasting patients changed their nutrition less frequent compared to the weight reduction diet patients and adhered seldom to learned nutritional aspects and to a lower-fat diet may be found by analysis of therapeutic approaches. While the weight reduction diet patients used the learned theoretical nutritional aspects practically during the whole inpatient therapy, the fasting patients collected after the passive state of food abstinence only few and short practical experience (3-4 days) of the possible modifications to their diet.

With regard to this possible explanation, a review that compares different approaches to the treatment of obesity shows that participants of study arms in 28 included studies that exclusively obtained a theoretical guidance for diet had similar results of long-term weight reduction as the fasting patients in the present study. A long-term weight loss over several years could not be reached by theoretical instruction [24].

The assumed potential of fasting therapy for long-term changes in diet [25] was not confirmed by the present study. The fasting therapy carried out as part of the inpatient naturopathic complex treatment in this patient population proved to be less suitable compared to standard therapy for the treatment of overweight and obesity.

Both the fasting and the weight reduction diet patients who increased their sports activity permanently showed no greater weight loss than those participants who did not or only for a short period increase their sports activity. A possible explanation is the lower energy costs for the sports activities compared to the increase in base and leisure activity. The sports activities were increased only for 2.0 hours per week (median) and most often in the form of gymnastics, a sport activity with comparable low energetic intensity. Levine showed in his review that people who reported to be physically active spent mostly no more than 2 hours per week for this and that this accounts only for an additional energy expenditure of 100 kcal per day [26]. However, this author showed also that a regular increase of the daily everyday life and leisure activity leads to a significantly higher energy expenditure and plays thus a crucial role in the regulation of overweight and obesity [26]. Therefore, the significant increase of base and leisure activity in weight reduction diet patients compared to fasting patients is another possible explanation for the successful long-term weight loss of weight reduction diet patients.

16% of the fasting and 28% of the weight reduction diet patients experienced a continued improvement in quality of life after discharge from hospital. Almost half of the fasting patients (44%) and 19% of the weight reduction patients stated that quality of life has improved in this regard only for a period (Figure 7). This period lasted for 10.0 months (median) in the group of fasting patients and for 11.0 months (median) in the group of weight reduction diet patients.

With respect to the 2.25 kg weight loss criterion according to Williams et al. [19] about half of the patients (approximately 59% of fasting patients and almost 50% of weight reduction diet patients) in this study, who could stabilize permanently a weight reduction of at least 2.25 kg, also specified a lasting improvement in the quality of life with regard to body weight.

While in the present study fasting and weight reduction diet patients considered their weight-related quality of life during the inpatient treatment equally, at the time of the telephone interview the weight reduction diet patients were significantly less affected (*P* = 0.033).

Thus even a relatively small reduction in weight may influence considerably the quality of life.

Fasting patients benefited less than weight reduction diet patients in terms of long-term weight loss as well as in terms of weight-related quality of life.

## 5. Conclusions

Fasting therapy in the context of inpatient naturopathic complex treatment is less suitable for the treatment of overweight and obesity than low-calorie diet. Fasting therapy followed by long-term supervised weight reduction diet and fasting therapy for the prevention of obesity remains to be evaluated in the future.

## Supplementary Material

The Supplementary Material shows the original questionnaire along with an English translation.

## Figures and Tables

**Table 1:** Reasons for exclusion from the phone interview.

	Fasting patients	Weight reduction diet patients
No longer valid phone number and no entry in a public phone book	19 (24%)	6 (21%)
Not reached	6 (8%)	2 (7%)
Refused to participate	15 (19%)	5 (18%)
Unable to remember the situations in question sufficiently	5 (6%)	2 (7%)
Insufficient German language skills	2 (3%)	1 (4%)
Participation in other organized weight reduction programs in the meantime	19 (24%)	3 (11%)
Malignant disease	5 (6%)	2 (7%)
Already dead	4 (5%)	5 (18%)
Bedridden	1 (1%)	0
Undergone intestinal resection	1 (1%)	0
Hospitalization because of an acute stroke at the time of the telephone interview	1 (1%)	0
Moved abroad	0	1 (4%)
Development of dementia	0	1 (4%)

**Table 2:** Demographic and biometric characteristics of the study patients.

	Fasting patients	Weight reduction diet patients
Living situation		
Living in partnership	66%	66%
Single	25%	21%
Living in partnership with children	8%	9%
Living with children alone	1%	4%
Highest graduation		
Elementary school	69%	74%
Secondary school	21%	13%
University-entrance diploma	10%	13%
Working status		
Employed	21%	28%
Housewives/unemployed	12%	6%
Pensioner	67%	66%
Gender		
Female	84%	77%
Male	16%	23%
BMI	30.8 ± 4.6 kg/m²	31.6 ± 4.7 kg/m²
