# Supplementary material for: Retrospective Long-Term Comparison of Naturopathic Fasting Therapy and Weight Reduction Diet in Overweight Patients
Source: Evid Based Complement Alternat Med. 2014 Jul 13;2014:453407. doi: 10.1155/2014/453407 (PMC4122067; doi:10.1155/2014/453407)
Supplement: Supplementary file 1 — The Supplementary Material shows the original questionnaire along with an English translation. [file 453407.f1.docx]

Fragebogen für die Fastenpatienten

***Questionnaire for fasting patients***

Klinik Blankenstein

***Blankenstein Hospital***

Bochum Ruhr-Universität

***Ruhr-University Bochum***

Fragebogen zu den Auswirkungen des Heilfastens

***Questionnaire concerníng the effect of therapeutic fasting***

In diesem Fragebogen geht es um Ihre damalige Heilfastentherapie in der Klinik Blankenstein.

***This Questionnaire deals with your former therapeutic fasting at Blankenstein Hospital.***

Bitte beziehen Sie Ihre Antworten nur auf das damalige Heilfasten und nach Möglichkeit nicht auf Einflüsse durch andere Anwendungen in der Klinik.

***Please refer only to your former therapeutic fasting and, if poassible, not to other applications in the hospital.***

Falls Sie eine Frage nicht beantworten wollen oder können brauchen Sie diese nicht zu beantworten.

***If you don´t want or cannot answer a question, you don´t need to answer.***

Ihre Antworten werden streng vertraulich gehandhabt und entsprechend des Datenschutzes in anonymer Weise ausgewertet.

***Your answers are strictly confidential and will be evaluated anonymously according to the rules of (German) data security laws.***

Wählen Sie für jede Frage die für Sie zutreffende Antwortmöglichkeit aus. Wenn die Antwortmöglichkeit mit einer Zeitangabe verbunden ist ergänzen Sie diese. Ist bei einer Frage eine Mehrfachauswahl von Antwortmöglichkeiten erlaubt, wählen Sie alle die für sie zutreffenden Antwortmöglichkeiten aus. Falls keine Antwortmöglichkeit gegeben ist, beantworten Sie die Frage frei.

***Chose for every question the response option appropiate for you. If the response option refers to a time specification add it please. If a question allows for multiple selection please chose those which apply to you. If there is no preformed answer please answer with a word or a sentence***

******

Die ersten Fragen beschäftigen sich unmittelbar mit dem damaligen Heilfasten.

***The first questions concentrate on the former therapeutic fasting.***

1. Welche Bedeutung hatte für Sie eine Gewichtsabnahme zum Zeitpunkt des Heilfastens?

***Was the loss of weight important to you when you were fasting?***

Für Sie war eine Gewichtsabnahme ...

***For you loss of weight was ...***

sehr wichtig

***very important***

wichtig

***important***

weniger wichtig

***less important***

unwichtig

***not important***

2. Wie bewerten Sie das damalige Heilfasten ?

***How do you valuate the former fasting***

Mit dem Heilfasten waren Sie ...

***Concerning the therapeutic fasting you were ...***

sehr zufrieden

***very satisfied***

zufrieden

***satisfied***

weniger zufrieden

***less satisfied***

unzufrieden

***dissatisfied***

3. Hatten Sie durch das Heilfasten Nebenwirkungen ?

***Did you suffer from adverse effects of therapeutic fasting ?***

(z.B. Kopfschmerzen, Schwindel, Krämpfe, Herzklopfen, Schlafstörungen, ...)

***(for example head ache, dizzyness, spasms, palpitations, sleep disturbance, ...)***

falls nein, weiter mit Frage 4 nein

***if no, continue with question 4 no***

falls ja ja

***if yes yes***

a. Welche Nebenwirkungen sind bei Ihnen aufgetreten ?

***Which adverse effects occurred )***

Mehrfachauswahl möglich

***multiple selection possible***

Kopfschmerz

***head-ache***

Schwindel

***dizzyness***

starke Hungergegefühle

***excessive hungry feeling***

Muskelkrämpfe

***muscle spasms***

Herzklopfen

***palpitations***

Schlafstörungen

***sleep disturbance***

Muskuläre Schwäche

***muscle weakness***

Unruhigen Beine

***restless legs***

andere Nebenwirkungen

***other adverse effects***

4. Wie empfanden Sie die Gewichtsabnahme, die durch das Heilfasten damals in der Klinik erreicht wurde ?

***How did you percieve the weight loss caused by therapeutic fasting at that time at hospital ?***

sehr zufriedenstellend

***very satisfying***

zufriedenstellend

***satisfying***

wenig zufriedenstellend

***less satisfying***

nicht zufriedenstellend

***unsatisfying***

******

Die folgenden Fragen beschäftigen sich mit der Entwicklung Ihres Körpergewichtes nach dem Heilfasten

***The following questions deal with your body weight after therapeuting fasting***

5. Haben Sie anschließend an das Heilfasten zu Hause weiter an Gewicht abgenommen ?

***Following fasting did you continue reducing weight at home?***

Falls nein, weiter zu Frage 5b. nein

***if no, continue with question 5b no***

falls ja ja

***if yes yes***

a.1. Wieviel haben Sie zu Hause weiter abgenommen ?

***How much did you lessen your weight at home ?***

a.2. Über welchen Zeitraum haben Sie weiter abgenommen?

***During which space of time did you continue weight reduction ?***

a.3. Haben Sie das Gewicht anschließend gehalten ?

***Did you maintain the weight afterwards ?***

ja, für einen Zeitraum von

***yes, for a space of time of***

ja, bis heute

***yes, until today***

nein

***no***

Bitte weiter mit Frage 6

***Please continue with question 6***

b. Sie anschließend an das Heilfasten zu Hause Ihr reduziertes Gewicht gehalten (d.h., nicht wieder zu genommen) ?

***Following fasting did you hold your weight at home (i.e., not increased again) ?***

ja, für einen Zeitraum von

***yes, for a space of time of***

ja, bis heute

***yes, until today***

nein

***no***

6. Wieviel beträgt Ihr aktuelles Gewicht ?

***What is your actual weight ?***

7. Wie empfinden Sie Ihr aktuelles Gewicht ?

***How do your rate your actual weight ?***

Mit Ihrem Gewicht sind Sie ...

***With your actual weight you are ...***

sehr zufrieden

***very satisfied***

zufrieden

***satisfied***

weniger zufrieden

***less satisfied***

unzufrieden

***dissatisfied***

******

Die folgenden Fragen beschäftigen sich mit Ihrer Ernährung

***The following questions deal with your nutrition***

8. Haben Sie Ihre Ernährung imAnschluss an das Heilfasten nach den Vorgaben der stationären Ernährungstherapie umgestellt ?

***Did you adjust your nutriton after fasting according to the inpatient nutritional therapy ?***

Falls nein/ja, für einen Zeitraum weiter mit Frage 9 nein

***if no/yes, for some space of time, continue with question 9 no***

ja, für einen Zeitraum von

***yes, for a space of time of***

ja, bis heute

***yes, until today***

a. Welche der folgenden Aspekte setzen Sie seit dem Heilfasten zu Hause um ?

***Which of the following aspects do you put into practice at home after fasting ?***

Mehrfachauswahl möglich

***multiple selection possible***

Sie verzehren ...

you eat ...

reichlich Brot, Nudeln, Kartoffeln und Reis

***abundant bread, noodles, potatoes and rice***

bevorzugt Vollkornprodukte

***preferentially whole grain product***

täglich Obst, Gemüse oder Salat

***daily fruit, vegetables or salads***

2-3 mal/Woche oder seltener Fleisch und Wurst und 1x/Woche Fisch

***two to three times a week or less meat or saucage an once a week fish***

bevorzugt fettarme Lebensmittel

***preferentially low fat food***

bevorzugt pflanzliche Fette

***preferentially plant fats***

bevorzugt kalorienarme Getränke

***preferentially low calory drinks***

Süßigkeiten, Kuchen, Gebäck selten und in Maßen

***Candies, cakes, pastries rarely and moderately***

Sie planen Ihre Mahlzeiten im Voraus z.B. für die folgende Woche

***You plan your meals in advance e.g. for the next week***

******

Die folgenden Fragen beschäftigen sich mit Ihrer körperlichen Aktivität in Alltag und Freizeit, jedoch nicht mit der Ausübung von Sportarten.

***The following questions deal with physical activity in everyday life and during leizure time, but not doing sports***

9. Haben Sie nach dem Heilfasten Ihre körperliche Aktivität im Alltag (z.B. Treppensteigen, Wege zu Fuß für Besorgungen) gesteigert ?

***Did you increase your physical activity in everyday life (for example climbing stairs, walking)?***

falls ja: für wie lange ja

***if yes: how long ? yes***

bis heute

***till today***

für einen Zeitraum von ___ körperlich aktiver und danach weniger

***for a period of time of ____ physical more activ and later less***

Falls nein nein

***if no no***

Sie waren auch schon vorher wenig aktiv

***you were little active before***

Sie waren auch schon vorher sehr aktiv und haben dies beibehalten

***you were very active before and maintained that***

Wie lange sind Sie durchschnittlich in einer Woche zu Fuß oder mit dem Fahrrad für alltägliche Aufgaben unterwegs z.B. zum Einkaufen, zur Arbeit oder ähnliches ?

***For how long per week do you walk or bike for everyday activities e.g. for shopping or going to work or similar ?***

Geben Sie die Zeiten in Minuten an. Falls Sie keine Zeit dafür verwendet haben, sagen Sie "Null"

***Give the time in minutes. If you did not spent any time on this state "zero"***

11. Steigen Sie regelmäßig Treppen

***Do you climb stairs regularly***

nein no

ja, ... Stockwerke ... mal am Tag

***yes, ... floors ... times every day***

12. Haben Sie einen Garten ?

***Do you own a garden ?***

falls nein, weiter mit Frage 13 nein

***if no, continue with question 13 no***

falls ja ja

***if yes yes***

a. Wieviele Stunden pro Woche arbeiten Sie im Garten

***How many hours do you work in your garden per week ?***

******

13. Haben Sie körperliche Freizeitaktivitäten wie Spazierengehen, Radfahren, Kegelen, Tanzen oder ähnliche vor dem Heilfasten ausgeübt ?

***Did you perform leizure time activities like walking, biking, bowling, dancing or something alike before the therapeutic fasting ?***

falls nein, weiter mit Frage 13b nein

***if no, continue with question 13b no***

falls ja ja

***if yes yes***

a. Haben Sie diese Aktivitäten nach dem Heilfasten vermehrt ausgeübt ?

***Did you increase these activities after the therapeutic fasting ?***

ja, bis heute

***yes, until today***

ja, Sie haben diese Aktivitäten für einen Zeitraum von ... vermehrt ausgeübt und danach mehr oder weniger damit aufgehört

***yes, you increased these activities for period of time of ... and stopped them more or less afterwards***

nein

***no***

b. Haben Sie nach dem Heilfasten begonnen, diese körperlichen Aktivitäten auszuüben ?

***Did you start these acivities after the therapeutic fasting ?***

ja, Sie üben diese Aktivitäten bis heute aus

***yes, you perform these activities until today***

ja, Sie haben diese Aktivitäten für einen Zeitraum von ... vermehrt ausgeübt und danach mehr oder weniger damit aufgehört

***yes, you increased these activities for period of time of ... and stopped them more or less afterwards***

nein

***no***

14. Wieveil Zeit pro Woche üben Sie diese körperlichen Aktivitäten (Spazierengehen, Radfahren, Kegeln, Tanzen oder ähnliche) aktuell aus ?

***How much time per week do you perform these physical activities (walking, biking, bowling, dancing or something alike) now ?***

Bitte geben Die die Dauer der Aktivitäten in Minuten oder Stunden/Woche an. Falls Sie keine dieser Aktivitäten ausüben, antworten Sie bitte "keine Aktivität".

***Please give the duration of these activities in minutes or hours per week . If you do not perform any of these activities, please answer "no activity"***

******

Die folgenden Fragen beziehen sich auf die Ausübung von Sportarten (z.B. Ballsport, Jogging, Schwimmen, Kraftsport, Wandern, usw.), welche regelmäßig ausgeführt werden - mit dem Ziel der Erhaltung oder Verbesserung der persönlichen Fitness.

***The following questions deal with performing sports (for example ball games, jogging, swimming, athletic sports, rambling, and so on), w2hich are performed regularely with the aim to preserve or improve the personal fitness.***

15. Haben Sie vor dem Heilfasten Sport getrieben

***Did you perform sports before the therapeutic fasting ?***

falls nein, weiter mit Frage 15b nein

***if no, continue with question 15b no***

falls ja ja

***if yes yes***

a. Haben Sie nach dem Heilfasten vermehrt Sport getrieben ?

***Did you increase sport activities after the therapeutic fasting ?***

ja, bis heute

***yes, until today***

ja, Sie haben für einen Zeitraum von ... vermehrt Sport getrieben und danach mehr oder weniger damit aufgehört

***yes, you increased your sport activities for period of time of ... and stopped them more or less afterwards***

nein

***no***

b. Haben Sie nach dem Heilfasten begonnen, Sport zu treiben ?

***Did you start sport acivities after the therapeutic fasting ?***

ja, Sie treiben bis heute Sport

***yes, you perform sport activities until today***

ja, Sie haben Sport für einen Zeitraum von ... getrieben und danach mehr oder weniger damit aufgehört

***yes, you increased sport activities for period of time of ... and stopped them more or less afterwards***

nein

***no***

16. Wieviel Sport treiben Sie aktuell pro Woche ?

***How much sports do you perform per week now ?***

Bitte geben Die die Dauer der Aktivität in Minuten oder Stunden/Woche an. Falls Sie keinen Sport treiben, antworten Sie bitte "keinen Sport".

***Please give the duration of sport activities in minutes or hours per week . If you do not perform any sport, please answer "no sport"***

******

Die folgenden Fragen behandeln die Lebensqualität in Bezug auf das Körpergewicht. Mit Lebensqualität sind vor allem körperliche Verfassung, psychisches Wohlbefinden, Kontakte zu anderen Menschen und die Fähigkeiten alltägliche Aufgaben auszuführen, gemeint.

***The following questions deal with quality of life in relation to body weight. Quality of life means primarily physical condition, psychological well-being, contact to other human beings and the ability to perform everyday duties***

17. Fühlten Sie sich vor dem Heilfasten durch Ihr Körpergewicht in Ihrer Lebensqualität beeinträchtigt ?

***Did you feel impaired in quality of life by your body weight before therapeutic fasting ?***

Sie fühlten sich ...

***Did you feel ..***

stark beeinträchtigt

***much impaired***

beeinträchtigt

***impaired***

kaum beeinträchtigt

***marginal impaired***

nicht beeinträchtigt

***not impaired***

a. Hat sich Ihre Lebensqualität bezogen auf das Körpergewicht nach dem Heilfasten erhöht ?

***Was quality of life related to body weight increased after therapeutic fasting ?***

ja, für einen Zeitraum von ...

***yes, for a period of time of ...***

ja, bis heute

***yes, until today***

nein

***no***

18. Fühlen Sie sich aktuell durch Ihr Körpergewicht in Ihrer Lebensqualität beeinträchtigt ?

***Do you feel impaired in your quality of life by your body weight now?***

Sie fühlen sich ...

***Do you feel ..***

stark beeinträchtigt

***much impaired***

beeinträchtigt

***impaired***

kaum beeinträchtigt

***marginal impaired***

nicht beeinträchtigt

***not impaired***

******

Bei den folgenden Fragen handelt es sich um Fragen zu Ihrer Person.

***The following are personal questions.***

19. Wie ist Ihre Lebenssituation ?

***What is your situation of life ?***

Sie leben allein

***You live alone***

Sie leben mit Partner

***You live with a partner***

Sie leben mit Partner und Kindern

***You live with a partner and children***

Sie leben mit Kindern

***You live with children***

Sie leben bei Ihren Eltern

***You live with your parents***

Andere Lebenssituation

***Other situation of life***

20. Welcher ist Ihr Schulabschluss ?

***What is your graduation ?***

Hauptschule

***Secondary modern school***

Realschule

***Junior high school***

Abitur

***University-entrance diploma***

Kein Schulabschluss

***No graduation***

21. Welches ist Ihr Berufsabschluss ?

***What is your training qualification ?***

Lehre/Berufsausbildung

***traineeship/apprenticeship***

Hochschulabschluss

***University degree***

Kein Berufsabschluss

***No apprenticeship***

22. Sind Sie berufstätig oder in Ausbildung ?

***Are you working or on training ?***

falls nein, weiter mit Frage 23 nein

***if no, continue with question 23 no***

falls ja, weiter mit den Fragen 22a. und 22b. ja

***if yes, continue with questions 22a. and 22b. yes***

a. Welche Tätigkeit beinhaltet Ihre berufliche Tätigkeit hauptsächlich ?

***What is your professional activity like ?***

Sitzende Tätigkeit (z.B. Büro)

***Sitting (for example office)***

Mäßige Bewegung (z.B. Hausmeister, Handwerker)

***Moderate exercise (for example concierge, craftsman)***

Intensive Bewegung (z.B. Postzusteller, Bauarbeiter)

***Intensive exercise (for example postman, builder)***

b. Welchen Beruf üben Sie aus ?

***Which job do you hold ?***

23. Beziehen Sie Rente

***Do you receive a pension ?***

falls nein, weiter mit Frage 24 nein

***if no, continue with question 24 no***

falls ja, ja, seit ...

***if yes,. yes, since ...***

a. Welchen Beruf übten sie aus ?

***Which job did you hold ?***

24. Welche Erkrankungen sind bei Ihnen nach der Entlassung aus der Klinik Blankenstein neu aufgetreten ?

***Which diseases did occur newly after your discharge from Blankenstein hospital ?***

25. Welche Medikamente nehmen Sie aktuell ein ?

***Which medicaments do you take now ?***

26. Haben Sie übergewichtige Familienmitgleider in Ihrer Blutsverwandschaft z.B. Ihre Eltern oder Geschwister ?

***Are there obese family members in your sibship, for example your parents or brothers and sisters ?***

ja

***yes***

nein

***no***

27. In welchem Lebensalter wurden Sie zum ersten Mal übergewichtig ?

***What age did you become obese for the first time ?***

28. Gibt es Faktoren, die Ihnen in den vergangenen Jahren seit dem Heilfasten die Gewichtsabnahme erschwert haben ?

***Are there factors hampering you to reduce weight during the last years since the fasting therapy ?***

falls nein, brauchen Sie die letzte Frage nicht mehr zu beantworten nein

***if no, you do not need to answer the last question no***

falls ja, ja

***if yes,. yes***

a. Welche der folgenden Faktoren haben Ihre Gewichtsabnahme erschweert ?

***Which of the following factors did hamper your weight reduction ?***

Mehrfachauswahl möglich.

***Multiple selection possible.***

Ihre Familie oder Ihr Umfeld unterstützt Sie nicht

***Your family our your social contacts do not support you***

Ihr Alltag ist sehr stressig

***Your everyday life is very stressful***

Eine Krankheit hindert Sie, körperlich aktiv zu sein

***Disease deters you from physical activity***

Medikamenteneinnahme erschwert Ihnen die Gewichtsabnahme

***Medicaments impair weight reduction***

Sie haben chronisch Schmerznen und essen als Ersatz

***You suffer chronic pain and eat for compensation***

Es sind einschneidende Lebensereignisse aufgetreten (z.B. Todesfall in der Familie)

***Dramatic events happened in your life (for example death of family members)***

Sie Haben das Rauchen aufgegeben

***You stopped smoking***

Eine gesundheitsbewußte fettmoderate Ernährung schmeckt Ihnen nicht

***Healthy nutrition reduced in fat is not tasty to you***

Es fällt Ihnen schwer, nur selten Süßwaren und Kuchen zu essen

***It is difficult for you to eat only rarely candies and cakes***

Andere Faktoren

***Other factors***

Vielen Dank für Ihre Mitarbeit

***Thank you for your participation***
